# The effects of trade liberalization on inequality in nutrition intake: empirical evidence from Indian districts

**DOI:** 10.1186/s12889-024-18749-7

**Published:** 2024-05-15

**Authors:** Yali Zhang, Saiya Li

**Affiliations:** 1 Key Laboratory of Land Surface Pattern and Simulation, Institute of Geographic Sciences and Natural Resources Research, Chinese Academy of Sciences, Beijing, 100101 China; 2 University of Chinese Academy of Sciences, Beijing, China

**Keywords:** Trade liberalization, Inequality, Macronutrient intake, Micronutrients intake, FAO/WHO GIFT

## Abstract

**Background:**

Despite the positive impact of trade liberalization on food availability in India, severe inequality in nutrition consumption at the district level persists. Empirical evidence on the relationship between trade liberalization and nutrition consumption inequality often offers a country-level perspective and generates disputed outcomes. The study aimed to explore the effects of trade liberalization on inequality in nutrition consumption at the district level in India and to examine the heterogeneity of the impact on different nutrition consumption.

**Methods:**

Our study employed the Gini Index to measure nutrition consumption inequality of 2 macronutrients and 5 micronutrients at the district level in India during 2009–2011, utilizing the comprehensive FAO/WHO individual food consumption data. The import tariff was adopted as a proxy for trade liberalization, as its externally imposed nature facilitates a causal interpretation. We further identified the direct causal relationship between food trade liberalization and inequality in nutrition consumption using a fixed effects model.

**Results:**

The results show that more than 50% of the individuals in the survey districts did not meet the dietary standards for both macronutrients and micronutrients. Food trade liberalization hindered the improvement of inequality in nutrition consumption. As import tariffs were reduced by 1%, the inequality in intake of calories, zinc, vitamin B1, and vitamin B2 increased significantly by 0.45, 0.56, 0.48, and 0.66, respectively, which might be related to food market performance. The results also highlight the positive role of the gender gap, female-headed households, and caste culture on inequality in nutrition consumption in India.

**Conclusions:**

To ease the shock of liberalization and minimize its inequality effects, complementary measures should be adopted, such as improving food logistic conditions in poor areas, and nutrition relief schemes.

**Supplementary Information:**

The online version contains supplementary material available at 10.1186/s12889-024-18749-7.

## Introduction

Currently, there are substantial inequalities in food consumption globally, which seriously hinders the achievement of Sustainable Development Goal 2—“By 2030, end hunger and ensure access by all people, in particular, the poor and people in vulnerable situations, including infants, to safe, nutritious and sufficient food all year round” [1]. Around 800 million people globally lack sufficient calorie consumption and more than 2 billion people suffer from micronutrient deficiency [2, 3]. At the same time, 2 billion people worldwide are over-consuming food [4]. Although the world currently produces sufficient food to meet the needs of all people, given the inequality of food consumption, we still live in a world with a double burden of malnutrition, especially in developing countries [5, 6]. Economic development levels, employment rates, population growth, and education levels are generally hypothesized as potentially important constraints to inequality in food consumption [7, 8]. In contrast, the impact of trade liberalization on inequality in nutritional consumption was not consistently concluded and is currently the focus of academic debate. Some scholars, by lumping data from developed and developing countries together, have concluded that food trade liberalization provided aggregate welfare gains for national food availability [9]. However, it has been argued that trade liberalization increases undernutrition if only focusing on developing countries [10, 11]. Developing countries’ inequality in nutritional consumption is typically pronounced with large vulnerable populations, owning limited resources compared to the wealthy. If food trade liberalization does not equitably increase food and nutritional consumption among the poor, the aggregate benefits of trade openness may be realized at a substantial social cost in terms of increased inequality. Due to frequent global food trade and the dramatic change of trade patterns, it is critical to quantitatively explore the impact of food trade liberalization on inequality in nutritional consumption, to design effective trade policy interventions to ensure access to nutritious and adequate food for all.

Trade liberalization indirectly influences nutritional consumption inequality through various pathways. Income inequality plays a crucial mediating role in the relationship between trade liberalization and nutritional consumption inequality. Beyer et al. [12] explained how trade policies indirectly impact nutritional consumption by affecting income distribution, highlighting the intricate relationship between trade openness, wage inequality, and nutritional disparities. Trade liberalization generally leads to increased income levels, potentially alleviating poverty and positively affecting nutritional consumption [13]. However, Winters and Martuscelli [14] emphasized that the effects on impoverished households vary depending on specific trade policies and household income sources. For example, involvement in export sectors often brings benefits, while engagement in import-competing sectors may lead to losses, influencing nutritional consumption patterns based on income sources and sectoral employment. Similarly, trade liberalization may worsen wage disparities between skilled and unskilled labor, potentially impacting low-income families’ ability to afford nutritious food choices. Additionally, trade direction is crucial, with North-North and South-South trade typically reducing inequality, while South-North trade may exacerbate it. Naanwaab [15] suggests that trade between countries at similar developmental stages may not significantly impact nutritional consumption inequality, whereas trade between developed and developing countries may widen the gap. Furthermore, trade liberalization promotes the spread of highly processed, calorie-rich, nutrient-poor foods in developing countries, altering dietary patterns and worsening nutritional inequality. Blouin et al. [16] highlighted how the increased availability of such foods disproportionately affects low-income populations relying on affordable processed food options, exacerbating nutritional disparities. In conclusion, the complex interplay between trade liberalization, income distribution, trade direction, dietary patterns, and nutritional consumption underscores the importance of considering specific economic and social contexts when studying the effects of trade liberalization on nutritional inequality.

Besides the influence of socio-economic factors, food production, availability and accessibility are also key factors impacting the relationship between trade liberalization and nutritional consumption, with opposite results obtained, and thus has been the focus of scholarly debate, as evidenced by various studies. On the one hand, food trade openness not only directly increases food availability and diversity, but also indirectly affects people’s purchasing power, which reduces inequality in nutritional consumption [9, 17, 18]. Traverso and Schiavo [19] analyzed the evolution of trade in macronutrients (carbohydrates, fats, and proteins) in 71 low-income countries over the period 1996–2014. The findings suggest that trade openness has a positive impact on nutrient availability in low-income countries, which may improve inequality in nutritional consumption. In addition, some scholars have suggested that increased food availability with more trade openness leads to lower consumer prices, which reduces economic access for net food buyers and alleviates inequalities in nutritional consumption [20]. On the other hand, food trade liberalization lowers prices for food producers, affecting the incomes of farmers and rural households and thus worsening inequality in nutritional consumption [21]. Moreover, it exposes importing food countries to economic shocks and affects the stability of domestic food prices [22]. Mary [20] estimated the impact of food trade openness on hunger of 52 developing countries between 1990 and 2013. The results showed that a 10% increase in food trade liberalization would increase the prevalence of undernourishment by about 6%. Flachsbarth and Garrido [23] investigated the impact of international food prices on domestic markets in six Latin American countries at different levels of trade liberalization. The findings indicated that higher trade openness was associated with higher consumer food price indices, which reduces the purchasing power of poor households and increases inequality in nutritional consumption. Existing studies on the relationship between trade liberalization and inequality in nutritional consumption have focused on heterogeneous groups of countries with varying food market structures, distribution mechanisms, and policy interventions [24, 25]. As a result, the findings have been the subject of intense debate.

Food trade liberalization has the potential to increase the availability of certain foods or induce changes in dietary patterns [26, 27]. However, it is crucial to acknowledge that these foods may not be equally distributed among all individuals and may not necessarily be nutrient-rich. Relying solely on the perspective of food availability in assessing the effects of trade openness, without considering actual food consumption and utilization, could lead to misleading conclusions. It is important to directly measure the level of inequality in actual nutrition consumption. To address this gap, we utilize the FAO/WHO Global Individual Food Consumption dataset, which offers exceptional suitability for accurately investigating the inequality in individual nutrition consumption. This dataset provides more compelling evidence compared to existing indirectly calculated nutrition consumption data based on national food expenditure statistics. In addition, limited attention has been given to inequality in micronutrients (vitamins and minerals) consumption in the context of trade liberalization, excluding macronutrients (calories, fats, and proteins). The under-consumption of calories remains a critical issue in some countries, while inequalities in micronutrient consumption contribute to more widespread hidden hunger globally, with potentially significant long-term health consequences, which are often overlooked.

India presents a particular context in which to seek answers to the impact of trade liberalization on inequality in nutrition consumption. Firstly, given the inequality of food consumption, India’s dual burden of undernutrition and overnutrition has been well documented at the national, state, and district levels [28]. The cereal intake of the bottom 30% of the population continues to be much less than that of the top two deciles, and the latter also had better affordability and access to non-cereal items like fruits, vegetables, and meat products [29]. Second, India’s food trade liberalization, characterized by large, rapid, and externally imposed implementation of tariff reductions [27, 30], contributes to the causal interpretation of the findings. Therefore, based on the context of India’s trade policies and nutritional status, and drawing from existing literature, we hypothesize that an increase in trade openness in India may exacerbate nutritional consumption inequality across different socio-economic strata. Specifically, we hypothesize that higher levels of trade liberalization in India will lead to larger disparities in nutritional consumption. Aligned with our hypotheses, this study aims to measure inequality in nutrition consumption of 2 macronutrients and 5 micronutrients across 99 districts in India from 2009 to 2011 based on FAO/WHO global individual food consumption data. The direct causal effect between food trade liberalization and inequality in nutrition consumption will be estimated by adopting a fixed-effect model, controlling for the potential effects of economic, social, and caste factors. The results indicated that inequality in both macronutrient and micronutrient consumption was a concern at the district level in India. Food trade liberalization was hindering the improvement of inequality in nutrition consumption, that is, a 1% reduction in import tariffs would increase the inequality in calorie, zinc, vitamin B_1,_ and vitamin B_2_ intake, ranging from 0.45 to 0.66. In addition, gender equality and caste culture are also key to increasing inequality in nutrition consumption.

## Data and methods

### Data

**Fig. 1:**
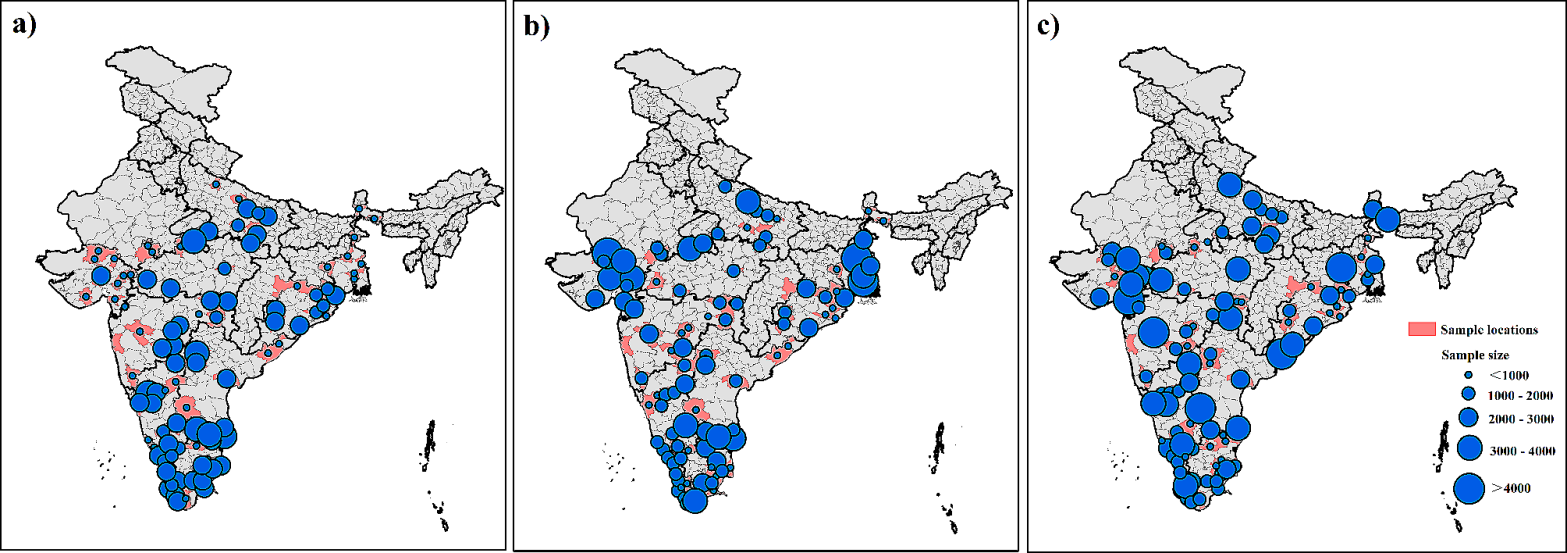
Sample distribution and number of FAO/WHO GIFT in India. **a**) - **c**) represent the years 2009, 2010, and 2011 respectively

FAO/WHO Global Individual Food consumption data Tool (FAO/WHO GIFT) is a novel open-access online platform [31], hosted by FAO and supported by WHO, providing access to harmonized individual quantitative food consumption (IQFC) data, especially in low- and middle- income countries (LMIC). FAO/WHO GIFT focuses on data collected through 24 h dietary recalls or records, which are tools describing in detail all foods and nutrients (macronutrients and micronutrients) consumed by individuals. This dataset offers the opportunity to calculate inequalities in nutritional consumption [32, 33]. The individual samples for FAO/WHO GIFT in India were distributed across 99 districts in 10 states, conducted in 2009–2011(Fig. 1). Although the coverage of individual samples of FAO/WHO GIFT from India is limited, GIFT adopted a rigorous sampling design, which usually takes into account factors such as population distribution, socio-economic characteristics, and food consumption habits in each region to ensure that the data are comprehensive and representative. Meanwhile, FAO/WHO GIFT used multiple sampling methods, including simple random sampling, stratified sampling, or multi-stage sampling, to ensure randomness and diversity of sampling, so that adequate and representative samples can be obtained from different regions and populations, thus reducing sampling bias. Thus, a rigorous sampling process enhances the reliability and validity of the findings, thereby contributing more effectively to the debate on trade liberalization and nutritional inequality in India. Using these data, we construct district-level metrics of inequalities in nutritional consumption for two macronutrients (energy and protein) and five micronutrients (iron, zinc, vitamin C, vitamin B_1_, and vitamin B_2_).

Other datasets include a variety of district-level economic and socio-demographic indicators and food production indexes. We use food import tariff data to measure India’s trade liberalization, derived from the World Integrated Trade Solution database (WITS). Other economic indicators, such as GDP and sectoral output, are sourced from the State Directorate of Economics and Statistics. The socio-demographic indicators cover mainly four aspects: demographic characteristics, educational characteristics, household characteristics, and caste characteristics, which are mainly obtained from census data. In particular, data on demographic characteristics include total population, sex ratio, the population aged 60+, urban or rural population, and literacy rate; data on educational characteristics include pre-primary education, primary education, secondary education, university education, and postgraduate education; data on household characteristics include the number of 1–2 person size households, 3–6 person size households, 7–9 person size households, 10–14 person size households, and female-headed households; and caste characteristics include scheduled tribes, scheduled castes, other backward, and other/upper castes. In addition, data on food production such as cereal production, fruit and vegetable production, meat production, egg production, and milk production are obtained from the State Directorate of Agriculture.

### Measurement and basic patterns of inequality and trade liberalization

Gini Index (GI) is chosen purposefully to measure the level of inequality considering its wide use, ease of computation, and comprehension. GI is a summary indicator for measuring inequality in society (varies from ‘0’ in case of perfect equality to ‘1’ [or 100 in percentage terms] in case of perfect inequality) [34]. The advantage of using GI considered the following principles: anonymity; population replication; scale invariance/mean independence/relative income and dalton transfer [35]. The inequalities in nutrition consumption at the district level were assessed using the following formula:

$$GI = \frac{1}{{2{{\rm{n}}^2}\mu }}\sum\limits_{j = 1}^{\rm{m}} {\sum\limits_{k = 1}^m {{n_j}{n_k}} } \left| {{y_j} - {y_k}} \right|$$

where *y*_*i*_ represents the consumption of nutrition items for the *ith* individual or in other terms, the per capita nutrition consumption of *ith* individual; then (*y*_*1*_, *y*_*2*_, …, *y*_*n*_) represents the distribution of consumption of nutrition items of the individuals *i* = *1, 2, … n* (or in other words, the distribution of per capita consumption on nutrition items of the population with n individuals). Inequality is the sum of all pair-wise comparisons of ‘two-individual inequalities’ that can conceivably be made. It is then normalized by dividing by *n* squared (because all pairs are added and there are *n*^*2*^ such pairs) as well as the mean monthly per capita nutrition consumption. The double summation signifies that we first sum over all *j*, holding each *k* constant, and then sum over all the *k*.

Considering India’s tariff reduction policy, characterized by a large, rapid, and externally imposed implementation nature, we use import tariffs of food productions to measure trade liberalization. Referring to the method of Montolalu et al. [36], tariff exposure is defined by weighting tariff lines according to the shares in each district’s regional GDP. We convert the national tariff of all food items to the district level. The equation is as follows:

$$Tarif{f_{d,t}} = \frac{{GRD{P_{d,t}}}}{{GD{P_{India,t}}}} \times {T_t}$$

where *Tariff*_*d, t*_ represents the tariff in each district *d* and period *t* and is generated from the national tariff *T* weighted by the GRDP (gross regional domestic product) in each district *d.*

## Empirical model and identification

We specified the model estimation to analyze the impact of trade liberalization on inequality in nutrition consumption. Meanwhile, several regression equations were established that varied based on different nutritional types. Based on the analysis of existing literature and to fulfill the purpose of our study, an empirical model using panel data fixed-effect regressions was constructed to determine the effect of trade liberalization on inequalities in nutrition consumption. The general form of the model is as follows:

$$N{I_{d,t}} = \alpha + \beta \cdot Tarif{f_{d.t}} + \sum\limits_{k = 1}^K {{\partial _k}} {X_{d,t,k}} + {\varepsilon _{d,t}}$$

where *NI*_*d, t*_ is district-level inequalities in nutrition consumption such as measures of inequality, and *Tariff*_*d, t*_ is the district exposure to international trade. The coefficient of interest, *β*, captures the average effect of trade openness on regional inequalities in nutrition consumption. Index *X* refers to the set of control variables that may influence inequality; the selected control variables include GDP per capita, sectoral output, unemployment rate, food production, total population, sex ratio, aging, urbanization rate, illiteracy rate, female literacy rate, education level, household size, female-headed households, and caste. Additionally, we incorporated the Trade Freedom Index as an alternative proxy variable to conduct robustness checks. This index serves as a comprehensive measure designed to capture a variety of trade restrictions, including tariffs, quotas, implicit administrative restrictions, and controls on exchange rates and capital flows, offering a more encompassing gauge of the degree of trade liberalization [37]. The trade freedom index, as a component of the overall Economic Freedom Index, was extracted from the database of the Heritage Foundation [38].

## Results

### Descriptive statistics

**Fig. 2:**
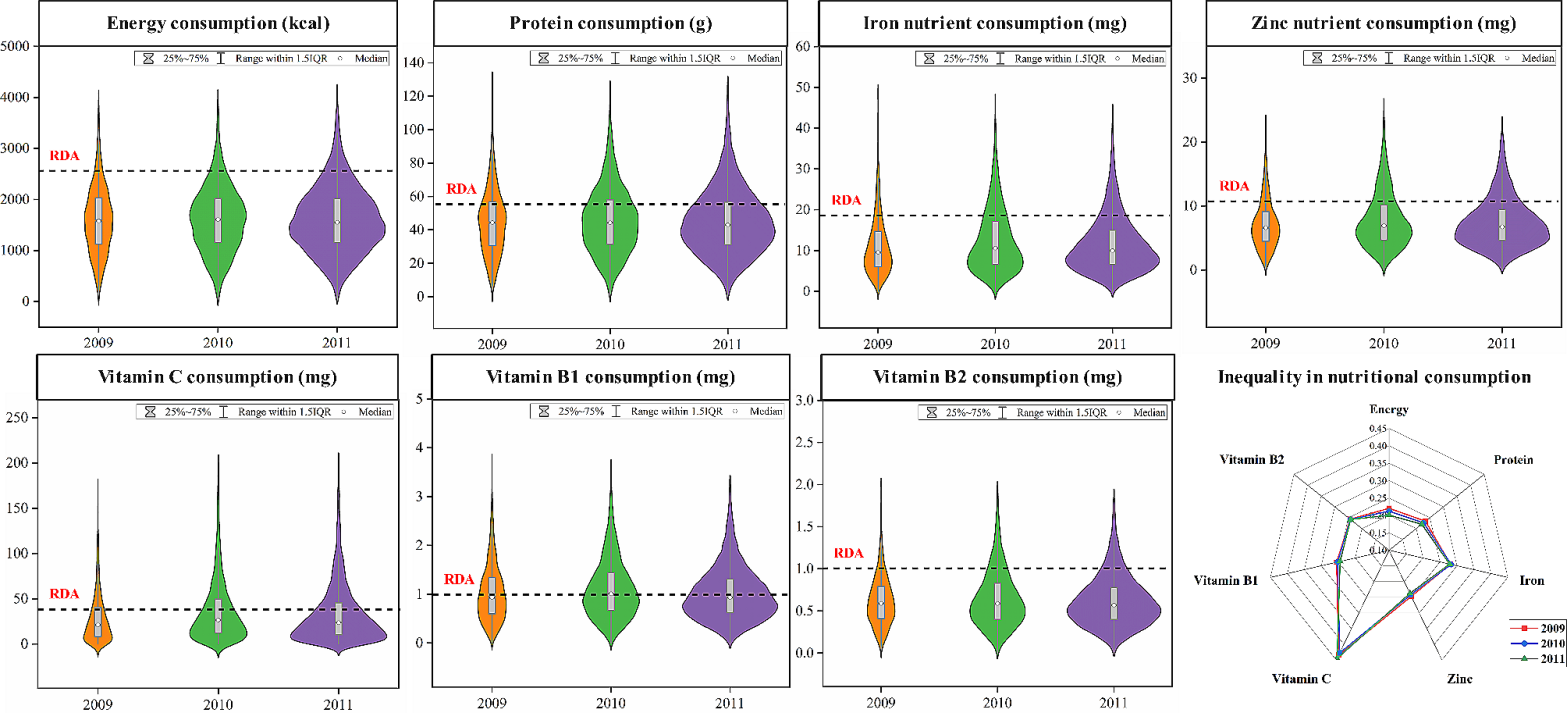
Distribution and inequalities in nutrition consumption, 2009–2011. The ICMR 2010 committee RDA recommendations revised and upgraded the RDAs for Indians based on the international data provided by FAO/WHO/UNU 2004 expert committee [44]

More than half of the Indians in the survey sample experienced both macronutrient and micronutrient deficiencies (Fig. 2). We refer to the Recommended Dietary Intake (RDA) indicators mentioned in the Indian Council of Medical Research (ICMR) and compare them with the distribution of the various types of nutritional consumption. For macronutrients, the average individual calorie consumption in the sample was much lower than the RDA, with approximately over 70% consuming fewer calories than the conventional ICMR norm, which is consistent with the results of Srivastava and Chand [39]. Individual protein consumption deficiency rates are lower than energy consumption deficiency rates, which is often explained as the “calorie consumption puzzle” [40]. Increasing incomes have led to increased economic access to food, leading consumers to reduce their intake of grains, but to diversify their diets and choose nutritious foods [29]. Compared to macronutrients, researches on micronutrient consumption are more limited. India has among the highest life expectancy losses due to micronutrient deficiencies [41, 42]. Our results indicated that more than two-thirds of the population consumed inadequate micronutrients, particularly iron and vitamin B_2_, and to a lesser extent zinc. These deficiencies are driven by the composition of the diet and the nutrient content of the main staple foods in India, such as rice and wheat [43].

Despite increasing food productivity since the successful economic reforms in India, inequality in nutritional consumption remains a major issue in the country [45]. The distribution of the seven categories of nutritional consumption was generally unequal (Fig. 2). The Gini Index (GI) of nutrition consumption was calculated to visualize the level of inequality in nutrition consumption. The results showed a decrease in inequality of energy and protein consumption between 2009 and 2011, from 0.22 to 0.20 and 0.23 to 0.22, respectively. This is a positive sign for the country, but we must point out that inequalities in calorie and protein intake may not reflect inequalities in the consumption of micronutrients such as minerals and vitamins, which are essential for health and affect the productivity of an individual [35]. Inequalities in the above items did not improve significantly during the study period. The highest level of inequality in vitamin C consumption was maintained, ranging from 0.42 to 0.44, followed by iron nutrients. The hotspots of inequality in micronutrient consumption were mainly in Kerala in southern India, and Maharashtra and Gujarat in western India, where inequality in nutrition consumption was generally higher than average.

**Table 1:** Descriptive statistics in the different survey years

Group	Variable	2009		2010		2011	
		Mean	Std. Dev	Mean	Std. Dev	Mean	Std. Dev
Trade	Import tariffs (%)	0.099	0.094	0.081	0.077	0.122	0.118
Economy	GDP per capita (millions in Rs)	47.737	25.530	56.432	29.672	63.818	34.264
	Primary sector shares (%)	23.635	11.280	24.838	12.189	24.372	12.359
	Secondary sector shares (%)	25.687	10.907	25.501	10.623	25.072	9.678
	Tertiary sector shares (%)	51.264	10.512	50.300	10.798	50.557	10.479
	Employment rate (%)	0.820	0.075	0.816	0.075	0.811	0.076
Food	Grain production (1000 tons)	554.887	435.316	666.300	528.423	676.122	512.632
	Fruit and vegetable Production (1000 tons)	452.703	456.877	364.546	435.535	371.310	411.323
	Meat production (tons)	8428.406	14418.96	9712.940	19952.3	13465.61	22460.48
	Egg production (Lakh number)	1158.186	2393.369	1171.969	2436.948	1149.577	2544.900
	Milk production (1000 tons)	217.658	182.024	245.096	192.638	261.870	216.377
Demographic	Total population (in thousands)	2692.663	1503.692	2722.614	1532.004	2752.564	1560.985
	Sex ratio	1.027	0.056	1.026	0.056	1.026	0.057
	Aging rate (%)	7.826	0.01838	8.624	0.02052	9.414	0.02286
	Urbanization rate (%)	29.589	0.18814	30.082	0.19186	30.567	0.19568
	Literacy rate (%)	65.387	0.10404	66.101	0.10241	66.807	0.10084
	Male literacy rate (%)	56.242	0.03512	55.978	0.03428	55.725	0.03352
	Female literacy rate (%)	43.758	0.03512	44.022	0.03428	44.275	0.03352
Education	Below primary (%)	18.060	0.06364	17.184	0.06421	16.391	0.06487
	Primary Education (%)	26.919	0.05419	26.759	0.05795	26.704	0.06219
	Secondary Education (%)	20.196	0.04276	20.502	0.04462	20.758	0.04647
	Higher Education (%)	26.719	0.06688	27.216	0.06963	27.624	0.07239
	Postgraduate Education (%)	8.105	0.03145	8.339	0.03294	8.524	0.03448
Household	1–2 person size (%)	15.004	0.03644	15.035	0.03672	15.062	0.03700
	3–6 person size (%)	70.392	0.06615	70.455	0.06630	70.510	0.06646
	7–9 person size (%)	12.753	0.07079	12.664	0.07105	12.586	0.07131
	10–14 person size (%)	1.851	0.01902	1.847	0.01904	1.842	0.01905
	Female-headed households (%)	8.747	0.069	8.059	0.063	7.499	0.059
Caste	Scheduled Tribes (%)	30.959	0.07731	31.172	0.07707	31.385	0.07688
	Scheduled Castes (%)	9.239	0.16756	9.219	0.16753	9.199	0.16750
	Other Backward Classes (%)	13.604	0.07310	13.537	0.07346	13.471	0.07382
	Other/UpperCastes (%)	46.192	0.13563	46.066	0.13573	45.940	0.13586
	N	99		99		99	

It was clear that India was moving at a slow pace in improving inequalities in nutrition consumption, which was associated with the economic, social, and food production factors that have been mentioned in previous literature [29]. Over time, each of these influences showed different characteristics (Table 1). India’s trade liberalization, characterized by large, rapid, and externally imposed implementation of tariff reductions, provides a useful opportunity to investigate its impact on inequalities in nutrition consumption. Import tariffs on food products at the district level in India were around 0.1% from 2009 to 2011. During the study period, India’s economy boomed and the average GDP per capita at the district level showed an increasing trend from Rs 47.74 million to Rs 63.82 million. The survey sample districts exhibited economic development dominated by the tertiary sector with a share of about 50%. India has also experienced increasing levels of food production at the district level, particularly for grains, meat, and milk. For socio-demographic factors, we noticed particularly gender inequalities, with more males than females in the survey districts and higher literacy rates for males than females. In addition, the level of education was mainly dominated by primary education and higher education, which accounted for more than 50%. Based on the difference in the ability of males and females to distribute food within the household [46], we focused on the factor of female-headed households, which accounted for approximately 8%. To capture the impact of caste cultural aspects, we counted four categories of castes according to the official Indian classification, with Other/Upper Castes being the most represented.

### Estimation results

The decline in tariffs as a result of trade liberalization appears to have exacerbated inequality in nutrition consumption. Our results indicated that trade openness showed a positive causal relationship with the inequality in nutrition consumption (Table 2). Ceteris paribus, as the food import tariffs decrease by 1%, on average, the inequality of calorie intake increased by 0.45, the inequality of zinc intake by 0.56, the inequality of vitamin B_1_ intake by 0.48, and the inequality of vitamin B_2_ by 0.66. Existing research suggested that increased food trade openness would logically increase the number of calories and nutrients available [9, 36]. However, our findings imply that those who need it most (living mostly in rural areas) may not benefit from increased food trade and that inequalities in nutrition consumption do not improve as a result. The reason may be related to national economic development levels, food availability, and food market performance [47]. We found that higher GDP per capita reduced inequality in calorie consumption, which conforms with previous empirical research based on other developing countries in the Asia region [48, 49]. In terms of different economic sectors, compared to the agricultural sector, increased output in the industrial sector significantly reduced inequality in calorie consumption and inequality in vitamin C intake, respectively; and the tertiary sector significantly reduced inequality in iron intake.

**Table 2:** Regression results of effects of trade liberalization on inequality in nutrition consumption

Independent Variables	Inequality of calories	Inequality of protein	Inequality of iron	Inequality of zinc	Inequality of vitamin C	Inequality of vitamin B_1_	Inequality of vitamin B_2_
**Tariff**	-0.450*	-0.248	0.165	-0.559**	-0.044	-0.480*	-0.659**
GDP per capita, logged	-0.086*	-0.053	-0.006	-0.092	0.022	-0.051	0.074
Sector (baseline: ‘Agriculture’ sector)							
Industry	-0.003*	-0.002	-0.001	-0.004	-0.002*	-0.003	-0.003
Tertiary	0.0002	-0.002	-0.001**	-0.003	-0.001	-0.001	-0.001
Employment rate	3.074	-2.004	-0.133	1.179	-0.273	2.145	1.708
Cereal production, logged	0.0004	-0.008	-0.009	-0.011	-0.007	-0.010	-0.024**
F&V production, logged	0.021	0.014	0.004	0.030	-0.009	-0.014	0.007
Meat production, logged	-0.008	-0.011***	0.002	-0.007	-0.009	-0.009*	-0.013**
Egg production, logged	-0.011*	0.003	-0.009**	0.005	0.008	0.001	0.005
Milk production, logged	0.009	-0.031	-0.025***	-0.017	0.011	0.005	0.026
POP, logged	1.036***	0.957***	0.024	0.989**	0.017	1.282***	0.573
Sex_ratio	-8.592***	-8.014***	-0.211	-6.129*	-0.239	-5.263**	-4.711
Aging rate	0.846	-0.363	-0.262	-0.922	0.641	-1.697	-3.131
Urbanization	0.994**	0.842*	-0.031	0.420	0.035	0.763	1.376*
Illiteracy rate	4.418*	5.339***	0.237**	6.699***	-0.172	8.833***	7.879***
Literacy rate (baseline: ‘Male’ literacy)							
Female Literacy rate	-16.316***	-16.552***	-2.014***	-19.579***	-0.124	-19.277***	-22.405***
Education level (baseline: ‘below primary’ education)							
Primary education	-5.643**	-0.451	0.052	-0.769	-0.182	-0.302	0.759
Secondary education	1.962	-0.717	0.111	0.803	-0.482	-1.062	-3.525
University education	-1.336	-1.332	0.725***	-2.819	0.465*	-1.832	-3.157*
Postgraduate education	-0.559	0.550	-0.530	2.664	-0.877	2.300	2.509
Household size (baseline: ‘1_2 person’ household size)							
3_6 person	7.225	5.854	-0.427*	3.596	-0.343	-0.960	-1.353
7_9 person	3.035	-7.842	-0.503	-0.694	0.780	-9.087	-6.096
10_14 person	-3.421	42.270	-0.002	-7.944	-2.376	14.516	-32.266
Female headed households	-3.603***	-3.200***	-0.071	-5.179***	-0.276	-4.621***	-3.377*
Caste (baseline: ‘Other/Upper’ caste)							
Scheduled Tribes	-7.119	-6.911	-0.086	-8.281	0.039	-3.251	-11.504
Scheduled Castes	-1.887	3.951	0.034	-2.350	-0.176	-6.729	-4.059
Other Backward Castes	1.904	3.487*	0.241**	0.397	-0.069	0.162	-0.262
Observations	170	170	170	170	170	170	170
R-squared	0.55	0.47	0.35	0.47	0.44	0.54	0.39

Standard errors in parentheses ***p < 0.01, **p < 0.05, *p < 0.1

Also noteworthy in Table 2 is the fact that the impact of food production factors on inequalities in nutrition consumption is not universal and only contributes to improving some inequalities in nutrition intake. The results showed that increased cereal and milk production only significantly reduced inequality in vitamin B_2_ intake and improved inequality in iron intake, respectively. In contrast, higher meat and egg production alleviated inequality in the intake of many nutrients, with meat production significantly decreasing inequality in protein consumption as well as improving inequality in vitamin B_1_ and vitamin B_2_ consumption. Potential explanations for the heterogeneity in the relationship between food production and inequality in nutrition consumption have two aspects. On the one hand, these differences are caused by divergent nutritional contents of food. On the other hand, evidence of linkages between food production and inequality in nutrition consumption occurs only in terms of food availability. Studies have shown that there are many interacting factors, covering food availability, accessibility, and affordability, which combined to determine individual nutrition intake choices [50].

Progress on improving inequality in nutrition consumption would also be hindered by social factors such as population growth, gender inequality, household-headed characteristics, and caste culture in India. Population growth can significantly increase inequality in nutrition intake of calories, protein, zinc, and vitamin B_1_. Moreover, inequality in nutrition consumption increases in the process of population urbanization, which lies mainly in the inverted U-shaped relationship between urbanization and inequality in nutrition consumption [51], with the initial stages of urbanization worsening inequality. Traditional patriarchal customs in India limit women’s access to resources and opportunities and exacerbate inequality in nutrition intake between the genders. Our results showed that a 1% rise in female literacy compared to male literacy significantly improved inequality in nutrition consumption ranging from 2.014 to 22.405. Meanwhile, given women’s advantage in intra-household resource allocation, a higher proportion of female-headed households can also significantly reduce inequality in nutrition intake. The caste culture in India is also a key factor in the inequality of nutrition consumption. We observed significant differences in inequality in nutrition consumption across caste groups. Compared to the ‘Other/Upper’ caste, a one unit increase in the ‘Other Backward’ caste was associated with a significant increase in inequality in protein and iron consumption of 3.487 and 0.241, respectively.

**Table 3:** Regression results of effects of trade freedom index on inequality in nutrition consumption

Independent Variables	Inequality of calories	Inequality of protein	Inequality of iron	Inequality of zinc	Inequality of vitamin C	Inequality of vitamin B_1_	Inequality of vitamin B_2_
**Trade freedom index**	0.587**	0.591**	0.245*	0.853**	0.202	0.646*	0.676*
Control variables	Yes	Yes	Yes	Yes	Yes	Yes	Yes
Observations	170	170	170	170	170	170	170
R-squared	0.54	0.48	0.35	0.46	0.44	0.53	0.36

Standard errors in parentheses ***p < 0.01, **p < 0.05, *p < 0.1

Food tariffs as a policy tool have a direct impact on the cost of food imports. This subsequently influences domestic market prices and consumer purchasing power, ultimately impacting nutritional consumption. To ensure the robustness of the findings, we introduced the trade freedom index as an alternative proxy for robustness testing. The trade freedom index is a comprehensive indicator that encompasses not only tariff levels but also non-tariff barriers, market access conditions, and trade facilitation measures, providing a more holistic measure of the degree of trade liberalization. The results showed a significant positive correlation between the trade freedom index and nutritional consumption inequality, indicating that an increase in the trade freedom index was associated with an increase in nutritional consumption inequality (Table 3). This finding also implies that trade liberalization may exacerbate nutritional consumption inequality. Meanwhile, population growth, gender inequality, household-headed characteristics, and caste culture likewise had significant effects on nutritional consumption inequality (Supplementary Table 1). The stability of the control variables indicates that the model is robust to external disturbances. By substituting the trade freedom index in our analysis, we confirm the general applicability of the link between trade liberalization and disparities in nutritional consumption.

## Discussion

We have systematically assessed the distributional characteristics of nutritional consumption at the district level in India during 2009–2011 and further empirically analyzed the role of trade liberalization on inequality in nutrition intake. We adopted the key data, i.e., FAO/WHO GIFT data, which provide an opportunity to comprehensively analyze the state of nutrition consumption and inequality in India from multiple perspectives, including macronutrients and micronutrients. Unlike existing studies on nutrition in India that primarily used the National Sample Survey dataset [52, 53], the FAO/WHO GIFT dataset records actual individual nutritional intake, providing higher accuracy and granularity in measuring nutritional consumption. In addition, our observed results empirically corroborate the local evidence of food trade liberalization and inequality in nutrition consumption in India. Inequality in nutrition consumption at the district level in India did not gain sufficiently from liberalization. Importantly, even if Indian population figures were to stabilize, it was unlikely that domestic production alone would be sufficient to close the current food gap [54]. In order to successfully address malnutrition, the gap between domestic production and food demand may have to be filled through increased imports. This implies that appropriate trade policy interventions will have to be made to optimize the volume of food imports and enhance food logistics in order to improve inequality in nutrition consumption.

One of the main barriers to eliminating inequality in nutrition consumption is the insufficiency of available dietary nutrition data to support effective evidence-based policies and programs. A growing number of national and subnational dietary nutritional consumption surveys have been completed in most countries worldwide over the past few decades. Individual-level quantitative dietary surveys based on retrospective or prospective methods, such as 24 h recalls or food records, provide disaggregated information on what people eat in a country. The combination of the quantities and frequency of foods and beverages consumed over a given period and linkage to food composition tables allows the calculation of energy and nutrient intakes [55]. However, nutrition intake data available from dietary surveys on large or small samples are often not harmonized and broadly accessible for use by researchers, policymakers, and other stakeholders. In comparison, the FAO/WHO GIFT data platform offers the most detailed inventory of individual quantitative nutrition intake surveys conducted in middle-income countries (e.g., India) to date [56]. Unlike NSSO data, which primarily capture household-level aggregates and rely on self-reported data, GIFT data offer higher granularity and accuracy in measuring nutritional consumption. The dataset records actual individual intake, enabling more precise analysis of macronutrient and micronutrient consumption patterns. Additionally, the dataset is collected using rigorous sampling procedures that ensure regional representativeness, capturing variability in nutritional consumption patterns across different districts. This approach complements existing studies using NSSO data and enriches the debate on food security and nutritional intake in India. Our study analyzed inequality in nutrition intake (e.g., macronutrients and micronutrients) using individual-level dietary nutrition data from India. This allows similar analyses to be carried out for any country, making it possible to have a better understanding of the state of nutrition inequality in the country and the opportunity to improve it. Therefore, the establishment of routine dietary nutrition surveys, with more expansive population and area coverage, together with data sharing and in-depth data analyses, are key to supporting policies to improve inequality in nutrition consumption.

Undernutrition and inequality in nutrition consumption at the district level in India were still severe, even in the face of high economic growth and improved agricultural production. India has witnessed high economic and agricultural growth in the past two decades. Agriculture recorded the highest growth from 2003 to 2012, coupled with marked improvement in grain production [57], which has transformed India into a surplus and net food exporter from a food deficit. Such macroeconomic performance would reduce the incidence of nutrition deficiency and inequality of intake. However, the realization of the growth went on contrary to the general expectation by increasing nutrition deprivation and driving inequality. Our results show that more than half of the population was experiencing macronutrient and micronutrient deficiencies (Fig. 2). Of these, the highest incidence of inadequate calorie intake was known as the “calorie consumption puzzle”. Possible explanations for this could be that as the economy boomed and per capita income levels improved, the need for calories declined and people tended to diversify their diets in favor of nutritious foods such as milk, meat, fruit, and vegetables [58]. In addition, inequality in Indian nutrition consumption did not improve significantly during the study period (Fig. 2). The trickle-down effects of economic growth and improved agricultural production on socio-demographic disadvantaged groups have not been realized. The high economic and agricultural growth in India had no positive impact on the weaker economic groups, which meant that inclusive growth was not achieved [59, 60]. Trade, social, gender, and caste are also key barriers to achieving inclusive growth and to improving inequality in India.

We innovatively demonstrated the direct causal relationship between trade liberalization and inequality in nutritional consumption by measuring district-level inequality in nutrition intake in India. There was some evidences of the negative impact of trade liberalization on inequality in nutrition consumption, but the relationship is mostly inferred indirectly in terms of food availability [20, 61], which blurs the specific food-nutrition linkages. Our study attempts to directly explore the impact of trade liberalization by measuring inequality in nutrition consumption, as well as refining the measurements of different types of nutrition consumption. The results suggested that reductions in tariffs due to trade liberalization would exacerbate inequality in the consumption of calories, zinc, vitamin B_1,_ and vitamin B_2_ (Table 2). Food trade liberalization is often considered as a core element aiming to improve national malnutrition [36]. However, most developing countries are not competitive enough to take advantage of the positive impact of trade liberalization on nutrition consumption, which is closely linked to income distribution, the performance of national food markets, etc. Income inequality plays a crucial mediating role in the relationship between trade liberalization and nutritional consumption inequality. Trade liberalization may worsen wage disparities between skilled and unskilled labor, potentially impacting low-income families’ ability to afford nutritious food choices. Moreover, the increased food supply under food trade liberalization may affect food producer price declines and thus affect the incomes of farmers and rural households [62, 63]. The gains from trade may be captured mainly by intermediaries in the food chain and may not reach food producers and rural areas. This mechanism is particularly likely in developing countries, where food markets have serious imperfections [24]. In addition, local prices of the most favored foodstuffs may rise with free trade, resulting in lower purchasing power for the weaker economic groups and leading to nutrition deprivation [64, 65].

India’s pronounced regional variations in economic development, agricultural reliance, and infrastructural adequacy play a critical role in determining the effects of trade liberalization on nutritional inequality. Economically robust regions, especially in western and southern India [66], often benefit from diversified economies. These diversified economies provide some protection against the fluctuations in agricultural pricing. As a result, they enhance employment prospects and improve access to a diverse food supply [67], potentially improving nutritional inequality. Conversely, regions that are less economically developed and heavily dependent on agriculture encounter significant challenges in adapting to the shifting demands of global markets. Their limited capacity to diversify income streams and susceptibility to price volatility can exacerbate nutritional inequities. As households grapple with affording a nutritionally balanced diet, the situation is further complicated by escalating food prices associated with trade liberalization [68]. Agriculture-dependent areas are significantly impacted by trade liberalization due to the exposure to global market prices. This exposure introduces income instability, which can lead to amplified nutritional inequities. When crop prices decline, these instabilities compromise the purchasing power for nutritious foods, further exacerbating the issue. Moreover, the state of infrastructure and logistics in India exerts a substantial influence on how trade liberalization impacts nutritional inequality [69]. Areas with well-developed infrastructure can more effectively manage the transportation and distribution of food items, including perishable goods that are nutrient-dense. This leads to a more efficient and economical distribution network, enhancing the accessibility and affordability of nutritious foods [70]. In contrast, regions lacking adequate infrastructure contend with increased transportation costs and higher rates of food spoilage [71], which restrict the availability and affordability of nutrient-rich foods, thereby narrowing consumer options. In summary, to harness the potential of trade liberalization for improving nutrition, it is imperative to implement targeted interventions that address these regional disparities. These interventions include strategic investments in infrastructure, support for small-scale farmers, and social safety nets for vulnerable households to ensure equitable nutritional outcomes across India.

Indian caste culture has an ongoing negative influence on the realm of nutrition [72, 73], despite the affirmative policy actions taken in India. Consistent with existing research [46, 74], ‘Other Backward’ castes suffered disadvantages related to nutrition outcomes in comparison to the ‘Other/Upper’ castes (Table 2). It has been argued that caste-based inequality in nutrition consumption arises from the income levels of different castes [75]. The findings of Choudhury et al. [74] showed that the income gap for different caste groups largely explained the 50 g gap in fruit and vegetable consumption between upper and lower castes. Their discovery supports the notion that historical and ongoing affirmative action policies in India may ultimately have an equalizing effect on food consumption. As these policies are mainly centered around quotas for disadvantaged castes in educational institutions and public sector employment, they will promote income equality between castes, which in turn will help to reduce the gap in nutrition consumption. It also implies that income support interventions for backward caste groups are key to alleviating inequality in nutrition consumption. Thus, the current positive affirmative policies in education and employment in India will eventually balance the incomes of the castes in the long run. Interventions such as cash transfers targeting lower castes may be more effective in the short term.

The gender gap is also crucial to improving inequality in nutrition consumption in India. Despite numerous government measures to encourage gender equality, the gender gap still exists in India [76]. India’s global gender parity score is 0.48, which represents an “extremely high” level of gender inequality [77]. The lack of gender equality limits women’s access to resources and opportunities, which will hinder the process of eliminating inequality in nutrition consumption. Our results show that increased female literacy rates can significantly decrease inequality compared to male literacy rates. Also, an increase in female-headed households can help lower inequality in nutrition intake (Table 2). This phenomenon is potentially explained by the different intra-household resource allocation priorities of males and females. In India, females usually prioritize family food and nutrition because they look after children and do not prefer to spend on non-desirable items. However, their male counterparts spend more on non-desirable commodities such as alcohol and cigarettes [57]. Therefore, strengthening the role of women in families and in society at large is an important component of improving inequality in nutrition consumption, and addressing inequality of opportunity is key to achieving it. Providing women with equal access to services, resources, and infrastructure, such as health care, education, credit, food, sanitation, and communication tools, women can be sustained to use their potential, skills, expertise, knowledge, and passions, which contribute to addressing inequalities in nutrition consumption.

Our study provided a comprehensive evaluation of the characteristics of nutrition consumption at the district level in India during 2009–2011 and confirmed the negative impact of trade openness on inequality in nutrition consumption. However, there are some limitations in this study that can be further explored. Firstly, the observational data quantity utilized for empirical analysis in this study possesses potential limitations. Due to the limited sample of observations in this study, this may reduce the reliability and stability of the findings. Despite the relatively small sample size, the data obtained through the rigorous methodology and standards of the FAO/WHO GIFT increased the reliability and validity of the study results. Additionally, multiple regression equations were established based on different nutritional types in this study, and the results remained robust across various dependent variables. Secondly, individual food consumption data in India included a wide range of nutrition intake of each respondent throughout the day, but the samples were surveyed on different dates. Based on the individual daily nutrition intake data, statistics on inequality in nutrition consumption may contain time errors. Thirdly, data on food tariffs at the district level were not available, so we might be overestimating/underestimating trade liberalization indirectly by downscaling national tariffs to the district level. If more detailed import data were available at the district level, this might be a more sensible way to measure trade openness. Nonetheless, this study has contributed to the debate regarding the relationship between food import tariffs and inequality in nutrition consumption at the sub-national level, and highlights the need for governments to pay close attention to the distribution of food for vulnerable groups under food trade openness, as well as take pragmatic trade policy interventions to improve inequality in nutrition consumption.

### Policy implications

This study contributes substantiated evidence revealing that the liberalization of food trade amplifies nutritional consumption disparities within India. In light of these findings, India must devise targeted intervention policies concurrently with the implementation of food trade liberalization, aimed at ensuring equitable outcomes throughout the liberalization process. In the short to medium term, a comprehensive strategy can mitigate the adverse repercussions of uneven nutritional consumption arising from trade liberalization. Firstly, the government could establish precisely designed food subsidy programs that prioritize vulnerable demographics. These subsidies would function as a protective layer, preventing further marginalization of disadvantaged groups due to escalated food costs resulting from liberalization. For instance, India’s National Food Security Act (NFSA) aims to provide subsidized food to approximately two-thirds of the rural population and one-third of the urban population, which includes both cereals and pulses, ensuring access to basic food items for those most in need. Secondly, to stabilize essential food commodity prices and curb speculative pricing fluctuations, an enforceable regulatory framework could be established. Such a framework would systematically monitor and modulate price shifts, safeguarding consumers from drastic price shocks and guaranteeing access to affordable staple foods.

Taking a broader temporal perspective, it is strongly recommended that the Indian government make substantial investments in fortifying transportation infrastructure, particularly in socioeconomically deprived regions. Through constructing and enhancing road networks and transportation facilities in these areas, the government can significantly improve spatial integration within the food market. This spatial harmony is essential for facilitating smooth food movement from production hubs to consumption areas, consequently fostering the even allocation of food resources nationwide. The Pradhan Mantri Gram Sadak Yojana (PMGSY), for instance, aims to provide all-weather road connectivity to rural areas, which is crucial for improving access to markets and reducing post-harvest losses. Additionally, the government’s efforts to upgrade the agricultural value chain through the ‘Aatmanirbhar Bharat’ (Self-reliant India) initiative also emphasize the importance of infrastructure development. This initiative includes measures to modernize agriculture, strengthen the supply chain, and create new opportunities for farmers and entrepreneurs, which can indirectly contribute to reducing nutritional disparities.

Our research underscores the importance of adopting supplementary policies alongside food trade liberalization in India. These multifaceted strategies encompass the short to medium-term measures and long-term strategies. By embracing this diverse spectrum of policy interventions, India can adeptly tackle the challenges inherent in trade liberalization, thereby ensuring that nutritional consumption becomes progressively marked by equilibrium, inclusivity, and equity across all strata of society.

## Conclusion

Despite the rapid growth of agricultural production in India after economic reforms, the survey districts showed worryingly low average consumption levels for all types of nutrition, with more than 50% of the population not meeting traditional RDA standards in 2009–2011. At the same time, nutrition consumption is highly unequal, with inequality in vitamin C consumption being the highest. As hypothesized, nutrition consumption at the district level in India has not benefited from food trade liberalization. All other conditions being equal, when import tariffs are reduced by one unit, inequality in calorie, zinc, vitamin B_1_ and vitamin B_2_ intake all significantly increase, ranging from 0.45 to 0.66. This is mainly related to the food market performance in India. The findings suggested that many factors, including the gender gap, female-headed households, and caste culture, are also important sources of inequality in nutrition consumption. Overall, the study highlighted the nutrition situation in India and the impact of trade liberalization on nutrition inequality, which are important findings from a policy perspective. With the population of India gradually growing, it would be difficult to narrow the food gap by solely relying on domestic production and would have to depend on food imports. Therefore, as India pursues trade liberalization policies, it should emphasize vulnerable people’s nutrition consumption and formulate some trade interventions to alleviate the shock of trade liberalization.

### Electronic supplementary material

Below is the link to the electronic supplementary material.

Supplementary Material 1

## Data Availability

The datasets used and/or analysed during the current study available from the corresponding author on reasonable request. We used secondary data from the FAO/WHO Global Individual Food consumption data Tool. The FAO/WHO Global Individual Food consumption data Tool is available online at https://www.fao.org/gift-individual-food-consumption/data/en.
