# Supplementary material for: The effects of trade liberalization on inequality in nutrition intake: empirical evidence from Indian districts
Source: BMC Public Health. 2024 May 15;24:1317. doi: 10.1186/s12889-024-18749-7 (PMC11094889; doi:10.1186/s12889-024-18749-7)
Supplement: Supplementary file 1 — Supplementary Material 1 [file 12889_2024_18749_MOESM1_ESM.docx]

**The effects of trade liberalization on inequality in nutrition intake:**

**Empirical evidence from Indian districts**

**Yali Zhang^a,b*^, Saiya Li^a,b^**

*^a^ Key Laboratory of Land Surface Pattern and Simulation, Institute of Geographic Sciences and Natural Resources Research, Chinese Academy of Sciences, Beijing 100101, China;*

*^b^ University of Chinese Academy of Sciences, Beijing, China;*

*^*^ Corresponding author. Institute of Geographic Sciences and Natural Resources Research, Chinese Academy of Sciences, Beijing 100101, China.*

*E-mail: [zhangyali@igsnrr.ac.cn](mailto:zhangyali@igsnrr.ac.cn)*

**Supplementary Table 1. Regression results of effects of trade freedom index on inequality in nutrition consumption**

| **Independent Variables** | **Inequality of calories** | **Inequality of protein** | **Inequality of iron** | **Inequality of zinc** | **Inequality of vitamin C** | **Inequality of vitamin B1** | **Inequality of vitamin B2** |
| --- | --- | --- | --- | --- | --- | --- | --- |
| **Trade freedom index** | 0.587****** | 0.591****** | 0.245***** | 0.853****** | 0.202 | 0.646***** | 0.676***** |
| GDP per capita, logged | -0.101****** | -0.062 | -0.009 | -0.111***** | 0.014 | -0.068 | 0.052 |
| Sector (baseline: ‘Agriculture’ sector) | | | | | | | |
| Industry | -0.004****** | -0.003 | -0.001***** | -0.005***** | -0.002****** | -0.004 | -0.002 |
| Service | 0.0002 | -0.003 | -0.001****** | -0.003 | -0.001 | -0.001 | -0.001 |
| Employment rate | 4.762 | -0.004 | -0.128 | 3.429 | -0.253 | 3.969 | 0.533 |
| Cereal production, logged | 0.005 | -0.004 | -0.008 | -0.004 | -0.006 | -0.005 | -0.017 |
| F&V production, logged | 0.016 | 0.010 | 0.004 | 0.023 | -0.008 | -0.020 | 0.001 |
| Meat production, logged | -0.011***** | -0.013******* | 0.002 | -0.011***** | -0.010 | -0.013****** | -0.018***** |
| Egg production, logged | 0.013****** | 0.004 | 0.009***** | 0.007 | 0.008 | 0.004 | 0.008 |
| Milk production, logged | 0.013 | -0.030 | -0.026******* | -0.013 | 0.007 | 0.009 | 0.033 |
| POP, logged | 1.108******* | 1.011******* | 0.016 | 1.085******* | 0.0002 | 1.359******* | 0.669 |
| Sex_ratio | -7.620******* | -7.300******* | -0.231 | -4.839***** | -0.258 | -4.213***** | -3.411 |
| Aging rate | 2.880***** | 1.330 | -0.362 | 1.869 | 0.494 | 0.515 | -0.544 |
| Urbanization | 0.792***** | 0.638 | -0.043 | 0.126 | 0.012 | 0.541 | 1.142 |
| Illiteracy rate | 5.057****** | 5.818******* | 0.262***** | 7.552******* | -0.119 | 9.523******* | 8.726******* |
| Literacy rate (baseline: ‘Male’ literacy) | | | | | | | |
| Female Literacy rate | -13.833******* | -14.465******* | -2.086******* | -16.163******* | -0.218 | -16.576******* | -19.264******* |
| Education level (baseline: ‘below primary’ education) | | | | | | | |
| Primary education | -6.046****** | -0.848 | 0.085 | -1.349 | -0.092 | -0.743 | 0.290 |
| Secondary education | 2.256 | -0.521 | 0.126 | 1.185 | -0.443 | -0.745 | -3.118 |
| University education | -0.539 | -0.732 | 0.766******* | -1.755 | 0.551***** | -0.971 | -2.101 |
| Postgraduate education | -2.118 | -0.986 | -0.534 | 0.414 | -0.910 | 0.587 | 0.689 |
| Household size (baseline: ‘1_2 person’ household size) | | | | | | | |
| 3_6 person | 6.995 | 6.334 | -0.453***** | 3.591 | -0.393 | -1.160 | -2.106 |
| 7_9 person | 3.583 | -6.882 | -0.475 | 0.292 | 0.867 | -8.452 | -5.744 |
| 10_14 person | -3.421 | 33.661 | -0.110 | -23.742 | -2.701 | 1.683 | -48.103 |
| Female headed households | -4.166******* | -3.579******* | -0.052 | -5.910******* | -0.239 | -5.227******* | -4.154****** |
| Caste (baseline: ‘other’ caste) | | | | | | | |
| Scheduled Tribes | -9.873 | -8.913 | -0.089 | -6.465 | 0.039 | -6.223 | -15.204 |
| Scheduled Castes | -1.076 | 4.954 | 0.049 | -1.086 | -0.145 | -5.824 | -3.255 |
| Other Backward Classes | 2.937 | 4.192***** | 0.248****** | 1.743 | -0.063 | 1.273 | 1.157 |
| Observations | 170 | 170 | 170 | 170 | 170 | 170 | 170 |
| R-squared | 0.54 | 0.48 | 0.35 | 0.46 | 0.44 | 0.53 | 0.36 |

Standard errors in parentheses ***p < 0.01, **p < 0.05, *p < 0.1.
